# The impact of unscheduled gaps and iso-centre sequencing on the biologically effective dose in Gamma Knife radiosurgery

**Published:** 2021

**Authors:** Thomas Klinge, Marc Modat, Jamie R. McClelland, Alexis Dimitriadis, Ian Paddick, John W. Hopewell, Lee Walton, Jeremy Rowe, Neil Kitchen, Sébastien Ourselin

**Affiliations:** 1 Wellcome/EPSRC Centre for Interventional and Surgical Sciences (WEISS), Dept. Medical Physics and Biomedical Engineering, University College London, London, UK; 2 Centre for Medical Image Computing, Dept. Medical Physics and Biomedical Engineering, University College London, London, UK; 3 School of Biomedical Engineering & Imaging Sciences, King’s College London, London, UK; 4 Queen Square Gamma Knife Centre, National Hospital for Neurology and Neurosurgery, London, UK; 5 Green Templeton College, University of Oxford, Oxford, UK; 6 The National Centre for Stereotactic Radiosurgery, Royal Hallamshire Hospital, Sheffield, UK; 7 Victor Horsley Department of Neurosurgery, National Hospital Queen Square, UCLH Trust, London, UK

**Keywords:** Biologically effective dose (BED), radiosurgery, iso-centre sequencing

## Abstract

**Purpose:**

Establish the impact of iso-centre sequencing and unscheduled gaps in Gamma Knife® (GK) radiosurgery on the biologically effective dose (BED).

**Methods:**

A BED model was used to study BED values on the prescription iso-surface of patients treated with GK Perfexion™ (Vestibular Schwannoma). The effect of a 15 min gap, simulated at varying points in the treatment delivery, and adjustments to the sequencing of iso-centre delivery, based on average dose-rate, was quantified in terms of the impact on BED.

**Results:**

Depending on the position of the gap and the average dose-rate profiles, the mean BED values were decreased by 0.1% to 9.9% of the value in the original plan. A heuristic approach to iso-centre sequencing showed variations in BED of up to 14.2%, relative to the mean BED of the original sequence.

**Conclusion:**

The treatment variables, like the iso-centre sequence and unscheduled gaps, should be considered during GK radiosurgery treatments.

## INTRODUCTION

In the five decades, since the first radiosurgical treatment, the Leksell Gamma Knife® (GK) has become standard equipment in stereotactic radiosurgery (SRS) procedures [1, 2]. Over this time five commercially available models have been introduced, resulting in changes in the different variables associated with the scheduling and execution of the dose delivery. Since treatments are only described in terms of the total physical dose, differences in the time-domain of the dose delivery, where there has been a very significant change, have not been taken into account. The assumption is made that a GK unit delivers the dose as a single dose fraction. No allowances are made for the potential for repair of sublethal radiation damage during the variable treatment periods, an effect known to be exposure time dependent [3]. This assumption would imply that the total physical dose is sufficient to predict treatment outcomes.

Studies in the rat spinal cord, that were related to conventional radiotherapy and brachytherapy [4], have shown that there was a significant change in iso-effective dose when irradiation exposures were variably extended in time by longer than an acute exposure of a few minutes. This, importantly, included the treatment time frame associated with SRS [5]. This change in dose, for a given iso-effect, could be explained by a two-component sublethal damage repair model. The fast repair component, with a half-time in the order of 12 min, explains why a significant but variable amount of repair of sublethal radiation damage would be associated with any GK treatment. The update of the radiation unit to Perfexion™ (PFX) GK, replacing the earlier Models B and C, removed the requirement for manual collimator and set up changes which were very time-consuming. However, by adherence to the same prescription doses, the differences in treatment times were generally not considered when switching from the older to the newer GK model.

Several earlier studies [6, 7, 8] illustrated how, for multi iso-centre treatments, the biologically effective dose (BED) between patients decreases as a function of the overall treatment time even for those cases receiving the same total physical dose. Moreover, in the individual patient, the BED on the physical prescription dose iso-surface also varies as a consequence of the specific dose exposures to individual voxels, indicating that the varying dose contributions from the different iso-centres may also influence the BED for a fixed physical dose.

Recently, a clearly defined association has been demonstrated between BED values and effect for continuous treatments using a single iso-centre for trigeminal neuralgia, raising the prospect of BED prescribed treatment planning [9]. A greater understanding of the factors influencing the BED of multi iso-centre treatments is now required to provide guidance for the potential delivery of BED treatment planning for multi iso-centre treatments. A recent study has also shown BED to be associated with outcomes after multi iso-centre SRS for Acromegaly [10]. In this study, a simplified version of the standard BED model [11] was used to retrospectively estimate a single BED value for each treatment plan.

The current study was thus designed to demonstrate the effects and feasibility of considering the full BED distribution during treatment planning and its execution for multi iso-centre plans. While previous studies [6,7,8] have largely illustrated the inter-patient variability in the BED, the current work investigates the intra-patient variability that needs to be considered when moving towards BED based treatment planning. Importantly, the BED model not only takes into account the total dose but also the varying dose-rate throughout the treatment delivery, including both beam-on and beam-off periods. As a result, changes in the order of delivery of iso-centres, which are currently not taken into account in GK treatments, will lead to changes in the calculation of BED. This study was aimed at quantifying the impact this source of variation has on the BED for patients treated with the PFX GK. Such an understanding is an essential prior requirement for the development of treatment planning systems that take account of the BED. If the order of iso-centre delivery is shown to have a substantial impact on the BED, this will demonstrate that future work on BED optimised treatment plans must take the delivery sequence into account. In addition, since unscheduled interruptions in dose delivery can occur in multi iso-centre treatments, the impact of gaps and their positioning in the treatment was also investigated. There is a multitude of causes for such an interruption which could include medical reasons, patient discomfort, and re-imaging when using the thermoplastic mask and motion tracking system of the new Icon GK. Based on observations in some historical cases and because it is also approximated to a single fast half-time for the repair of sublethal damage a nominal gap duration of 15 min was used. Such unscheduled interruptions to a planned treatment would require adjustment to any proposed BED related treatment plan. The standard BED model with bi-exponential repair [4] was used in the analysis of a representative (in terms of the spread of overall treatment times) cohort of 15 patients treated for Vestibular Schwannoma using PFX using prescription iso-doses of either 12 or 13 Gy.

## MATERIALS AND METHODS

### BED model

For this study, the BED model developed by Millar and Canney [12], as revised by Pop et al. [4], was used. It takes account of the dose-rate variation throughout the treatment period, treating each iso-centre as a separate sub-fraction and the gaps in treatment between iso-centres as incomplete repair intervals. The fast and slow components of repair (µ_1 _and µ_2_) are combined in a partition model (partition coefficient: 
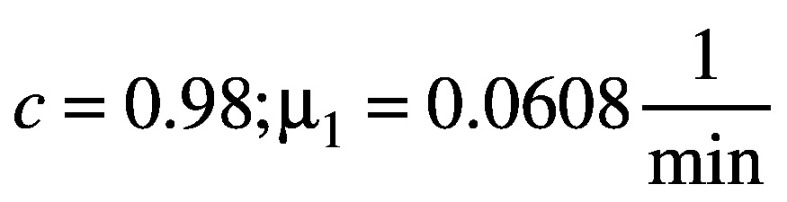
 and 
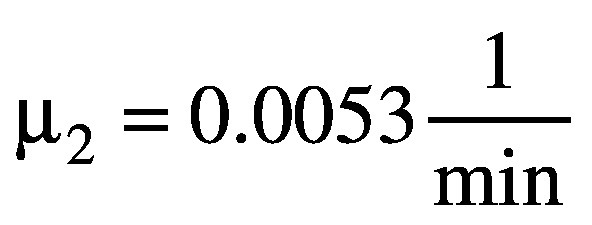
) using the equation:

**(1):**
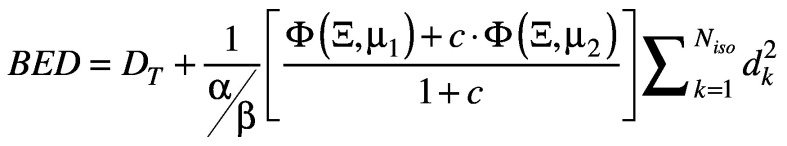


Where *D_T_* is the total dose delivered during the treatment, *N_iso _*is the total number of iso-centres (sub-fractions), *d_k _*is the dose delivered with iso-centre *k *(*k *∈ 1...*N_iso_*). The tissue parameters are α and β, and Φ(Ξ,μ) is a function of the treatment protocol Ξ, containing information about the timing and dose of every iso-centre. Since the in-patient dose-rate throughout the treatment varies from voxel to voxel, equation 1 has to be evaluated for every individual voxel in the region of interest.

### BED calculation for GK treatments

To enable BED calculations, using equation 1, a research version of Leksell GammaPlan® (LGP) version 10.1 was used to export the dose matrices and beam-on times for each iso-centre in the region of interest. The inter iso-centre (sub-fraction) times are manually extracted from the treatment protocol, assuming fixed gap durations between iso-centres (an average gap time of 0.06 min was estimated using the average beam-off periods from the current treatment protocols). The exported dose and timing information was processed in a MATLAB environment to calculate the BED distribution and to enable further detailed analysis.^†^ †MATLAB code to calculate BED from the per iso-centre dose distributions is available on GitHub: https://github.com/klinge-th/modelBED

When investigating the influence of unscheduled gaps in the treatment and the re-ordering of the iso-centre sequence, the mean BED on the prescription dose iso-surface was calculated, using all voxels within a dose range of ± 0.02 Gy of the prescription iso-dose. This dose range corresponded to dose deviations of < 0.16% and < 0.17% for the prescription doses of 13 Gy and 12 Gy, respectively. A similar approach has been adopted previously, e.g. [6, 7]. This approach allows for the comparison of the BED values for those voxels that are considered most representative of the dose prescription.

### Patient cohort

Patients included in the present study had been diagnosed with a Vestibular Schwannoma and treated with the PFX GK. In total, there were 15 cases treated in a single session with a prescription dose of 12 or 13 Gy, a total of 6 and 9 cases, respectively.

### Investigation

#### Overview of the BED for the original treatment

**Figure 1:**
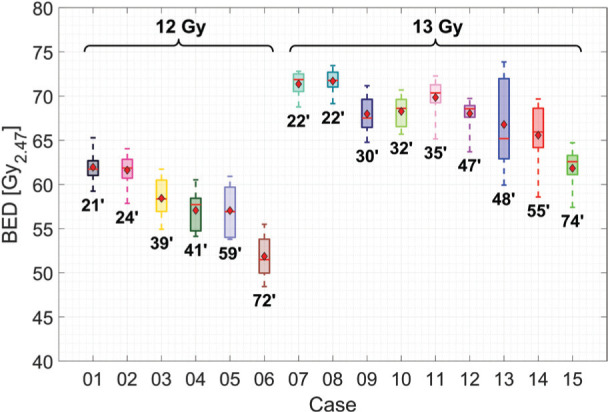
BED distribution for the individual cases on the iso-surface for treatments with prescription doses of 12 and 13 Gy with their respective total treatment times. The box plot shows the 25-75% quantile, the maximum, and the minimum BED. The median and mean BED values are marked by a red line and diamond marker, respectively.

Using the standard method described above, the BED distributions for the cohort of patients, as originally planned, were calculated first (see Figure 1).

#### Impact of an unscheduled gap in treatment delivery on the BED

To investigate the potential influence of unscheduled interruptions in the treatment delivery on the BED, a gap of 15 min was introduced for all possible locations between iso-centres. This was achieved by a 15 min increase to the otherwise short beam-off period required to reposition the patient. As discussed previously, this gap time is approximately one fast half-time for repair of sublethal radiation damage. Thus, for the purpose of BED calculations, the original planned total treatment times were all increased by 15 min.

#### Impact of changes to the sequence of delivery on the BED

In order to assess the influence of the re-ordering of iso-centres, for a given treatment plan, all deliverable permutations of the order of iso-centres originally prescribed were evaluated. For a given treatment with ‘*n*’ iso-centres, there are *n*! possible sequences in which it could be delivered.^‡^ ‡For n = 8 there are 8! = 40,320 possible combinations. For n = 13 this increases to ≈ 6.2270 × 109 sequences. Thus for practical reasons, only cases with a relatively small number of prescribed iso-centres (8 or fewer) were evaluated for all possible permutations to maintain reasonable computation times and avoid memory-related issues.

Based on the analysis of the iso-centre sequences, a heuristic approach was used to maximise and minimise the BED values for all patients in the cohort. This was achieved by grouping or distributing the order of delivery of the iso-centres according to their mean dose-rate at the prescription dose iso-surface. This mean dose-rate per iso-centre was chosen for selective purposes only since it correlated with the resulting mean BED value in the iso-centre sequence analysis (further details presented in the discussion). The resulting BED values serve as an estimation of the range of achievable BED values for a given treatment, solely by adjustments to the order of delivery of the different iso-centres selected originally.

## RESULTS

### Overview of the BED for the cohort based on the original treatment plan

For patients treated with the PFX GK, there was a variation in the range of BED values (individual patient mean and range) for cases as originally treated for both prescription iso-doses (see Figure 1). Since the cases are ordered according to increasing overall treatment time, the trend for a decrease in BED with increasing overall exposure periods is apparent [11]. In addition, it can be seen that 12 Gy in 20.8 min (13 iso-centres, mean BED: 61.95 Gy_2.47_) is approximately iso-effective (iso-BED) with 13 Gy delivered in 73.68 min (17 iso-centres, mean BED: 61.83 Gy_2.47_).

### Effects of gaps in the original treatment delivery

**Figure 2:**
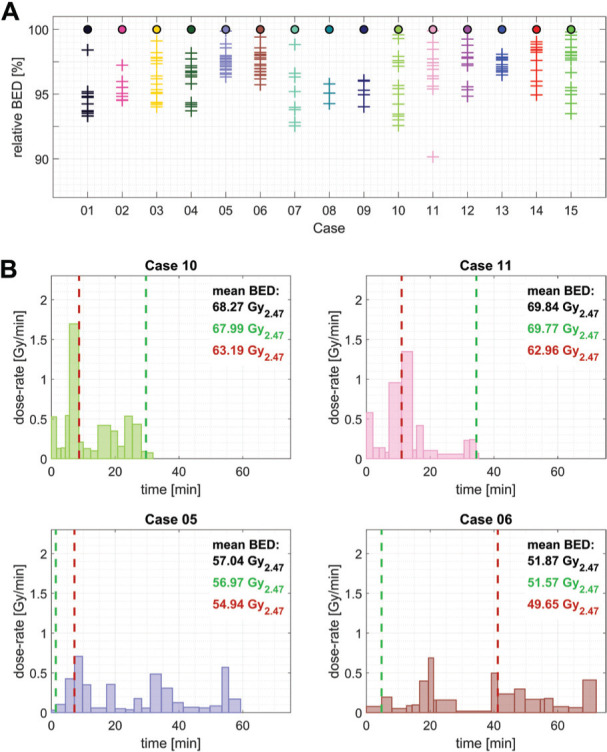
a) Relative change of the mean BED on the prescription dose iso-surface due to changes in the positioning of a single 15 min gap, variably positioned in the original treatment plan (see text). b) Mean doserate for voxels on the prescription dose iso-surface over the duration of the total treatment time for four of the fifteen PFX GK cases. The most and least impactful position for the 15 min gap, in terms of the relative reduction in BED, is marked with red and green dashed lines, respectively. The mean BED values for the original plan (black), the lowest impact gap (green), and the highest impact gap (red) are shown.

The relative reductions of the mean BED values for the prescription dose iso-surface, due to the introduction of a gap (15 min) into the original treatment protocol, between the different iso-centres, is shown in Figure 2 a). The magnitude of the effect clearly varies from patient to patient and with the position of the gap between the different iso-centres. The decrease ranges between 0.1 and 9.9%, indicating that knowledge of the existence of an unscheduled gap and its duration is insufficient to predict the outcome in terms of the reduction in biological effectiveness of the prescribed treatment.

Examples of treatments that exhibit either a high (top two panels) or low (bottom two panels) impact of the unscheduled gap on the average BED value are shown in Figure 2 b). The locations of that unscheduled gap with the least and greatest impact are marked on plots of the mean dose-rate per iso-centre versus the overall treatment time.

For a treatment where a large part of the total dose is delivered in one or a few high dose-rate iso-centres, a 15 min unscheduled gap in the dose delivery will have the highest impact at the temporal location of those iso-centres. Since the rate of induction of sublethal radiation damage is also proportional to the dose-rate, such a gap allows for the repair of sublethal damage before it can be translated into lethal damage by the dose from the next iso-centre, which would provide an explanation as to why splitting up two high dose-rate iso-centres with an unscheduled gap created the largest reduction in BED. If the mean dose-rate throughout the treatment is relatively low, a single unscheduled interruption in treatment has a smaller impact.

These observations are in agreement with the observation that the order in which iso-centres are delivered in an individual treatment influences the resulting BED distribution for that case.

**Figure 3:**
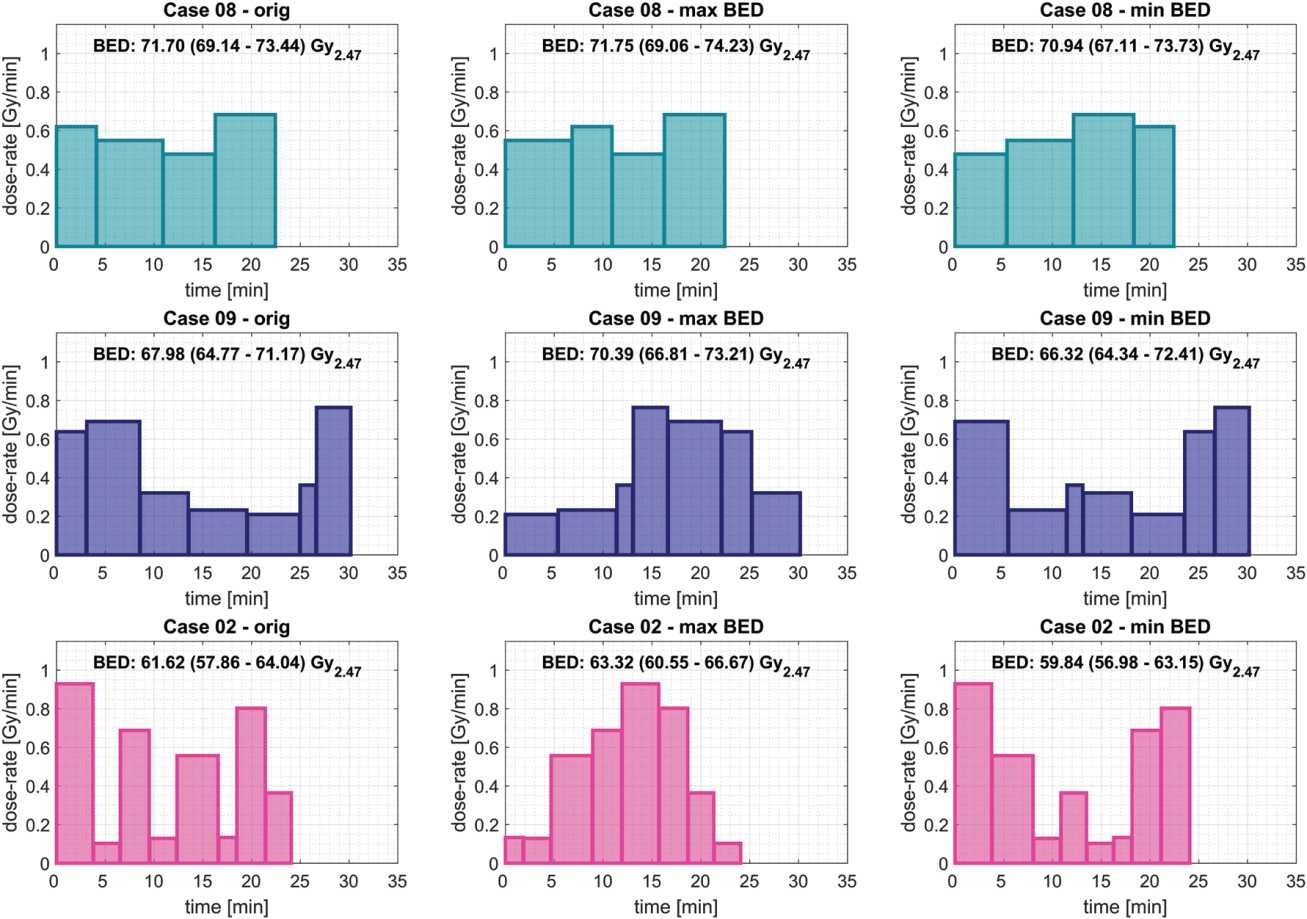
Mean dose-rate for voxels on the prescription dose iso-surface over the duration of the total treatment time for the original (left), ‘max BED’ (middle), and ‘min BED’ (right) sequences of the three cases investigated. The mean BED and range of values are shown for each case.

### Iso-centre ordering

For the three cases of this cohort of patients that involved less than 9 iso-centres, the BED distributions of all possible permutations of the sequence of delivery were calculated. From the up to 40,320 sequences per patient, those leading to the ‘max BED’ and ‘min BED’, expressed in terms of the mean BED on the prescription dose iso-surface, are identified. Figure 3 shows the original dose-rate profiles next to the ones now identified as ‘max BED’ and ‘min BED’ together with the associated mean and range of BED values.

**Figure 4:**
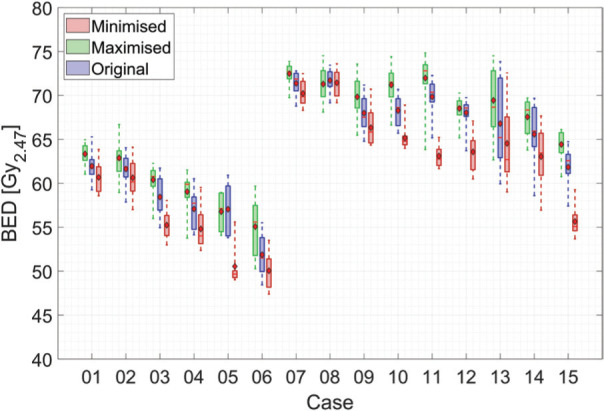
BED distribution on the prescription dose iso-surface for the original and optimised sequences of all cases in the present cohort. The boxplot shows the 25-75% quantile, the maximum, and the minimum BED. The median and mean BED values are marked by a red line and diamond marker, respectively.

The simplest 4 iso-centre plan (case 08) showed the smallest variation in the ‘max/min’ mean BED associated with shot ordering, namely 1.1% (range of mean BED values relative to the mean BED for the originally planned treatment sequence). This limited impact of the iso-centre ordering was perhaps to be expected since all 4 iso-centres had similar mean dose-rates delivered over a short period of time. For the other two cases, treated using either 7 or 8 iso-centres (cases 09 and 02), the variation observed was 6.0% and 5.6%, respectively. The dose-rate profiles of these sequences show that grouping the high dose-rate iso-centres together maximises the calculated mean BED value, whereas spreading out these high dose-rate iso-centres minimises the mean BED value.

Based on this observation, the iso-centre sequences for all cases were heuristically re-sequenced in order to achieve the maximum or minimum mean BED value on the prescription dose iso-surface. To achieve this, iso-centres were evaluated on the basis of the mean dose-rate, on the prescription dose iso-surface, and then the highest dose-rate iso-centres were either grouped together in a pyramidal shape (‘max BED’) or spread-out across the treatment delivery to have the maximum time between those same iso-centres (‘min BED’) as is illustrated in Figure 3 for cases 09 and 02, respectively.

For 13 of the 15 cases investigated in this illustrative cohort of cases, an increased mean BED value was observed when the proposed BED-maximising pyramidal sequencing approach was used (Figure 4). The only two cases that showed a decrease in BED after this ‘maximisation’ process were cases 08 and 05. Case 08 used only 4 iso-centres, as discussed previously, with no obvious pattern to the maximum BED sequence. For the other case (case 05), which showed a small decrease in mean BED, a visual assessment of the target geometry detected an elongated shape, along a single axis. This unique location of the individual iso-centres could not be accounted for using the heuristic approach. A comparison with the results in Table 1 showed that the range of observed BED values obtained was nevertheless comparable to the remainder of the cohort. To determine if there is a statistically significant difference, in terms of the mean BED on the prescription dose iso-surface, between the ‘min’ and ‘max’ sequences, a paired t-test was performed. Using the mean BED values of the two sequences for the ’12 Gy group’, the ’13 Gy group’, and the entire cohort together showed a highly statistically significant difference in all cases (p<<0.05, see Table 1 for precise values).

Generally, the range of achievable mean BED values scales as a function of the number of iso-centres originally used. The largest range of mean BED values obtained, relative to the original treatment, was 14.2% for a treatment involving the use of 17 iso-centres (the average across the illustrative cohort was 7.34%). Within this small cohort of patients, the variation between the smallest and largest observed mean BED values (across all patients) was 22.9% (50.0-63.3 Gy_2.47_) and 24.8% (55.6-72.5 Gy_2.47_) of the average original mean BED value, for the 12 Gy and 13 Gy prescription doses, respectively. The average increase in the mean BED achieved, using the heuristic approach, was 2.6%, whereas the average decrease was 4.7% (compared to the value for the original treatment sequence). The average BED value for 13 Gy case is 17% higher than for 12 Gy cases for original, ‘min’ and ‘max’ sequences.

## DISCUSSION

The results of the present study confirm that there was a large variability in the BED values for the treatment plans, as originally prescribed. For a given physical prescription dose, the BED progressively declines with total treatment time and different prescription doses can be iso-effective in relation to BED values (Figure 1). This finding for cases treated with the PFX GK is in agreement with previous studies using predominantly the Model B GK [6, 7, 8]. Reduced efficacy in the treatment due to unscheduled gaps is currently not taken into account. However, just knowing about the existence and duration of any unscheduled interruption in treatment delivery is clearly insufficient to estimate the true impact on the BED. The temporal position of that unscheduled gap, relative to the iso-centre sequence, has to be considered as well.

Analysing the impact of changes to the iso-centre sequences, compared to the original plan, also revealed another source of variability. The introduction of more small/heavily blocked (low dose/dose-rate) iso-centres into a treatment plan, e.g. to improve the conformity of the physical dose plan to the target [14], will at the same time also increase the total treatment time. This increase in treatment time will inevitably reduce the mean BED value associated with the selected prescription dose.

**Table 1:** Results of the heuristic sequence optimisation of the entire cohort of cases

case	dose [Gy]	T [min]	iso-centre number	mean BED [Gy_2.47_]			
				BED_orig_	BED_min_	BED_max_	Δ_rel_ [%]
01	12	20.80	13	61.95	60.67	63.33	4.29
02	12	24.07	8	61.62	60.63	62.88	3.67
03	12	39.06	17	58.44	55.24	60.39	8.82
04	12	41.00	14	57.07	54.80	59.04	7.42
05	12	59.32	19	57.04	50.54	56.77	10.93
06	12	72.05	19	51.87	50.05	55.07	9.68
**12 Gy average:**		**42.72**	**15.00**	**58.00**	**55.32**	**59.58**	**7.47**
**12 Gy t-test ‘min’ vs ‘max’:**				**statistically significant**		**p = 0.0011**	
07	13	22.37	9	71.37	70.22	72.47	3.15
08	13	22.44	4	71.70	71.43	71.29	-0.20
09	13	30.17	7	67.98	66.32	69.83	5.16
10	13	31.84	15	68.27	65.16	71.18	8.83
11	13	35.22	13	69.84	63.10	71.97	12.69
12	13	46.95	11	68.02	63.57	68.50	7.24
13	13	48.32	12	66.78	64.53	69.43	7.33
14	13	55.25	13	65.57	63.03	67.55	6.88
15	13	73.68	17	61.83	55.64	64.42	14.19
**13 Gy average:**		**40.69**	**11.22**	**67.93**	**64.78**	**69.62**	**7.25**
**13 Gy t-test ‘min’ vs ‘max’:**				**statistically significant**		**p = 0.0010**	
**Cohort t-test ‘min’ vs ‘max’:**				**statistically significant**		**p < 0.0001**	

Furthermore, a change in the order of the original iso-centre sequence also impacts the biological effectiveness of the treatment plan. Moreover, it is feasible to increase or decrease the marginal BED of the prescription iso-dose of a given treatment simply by re-arranging the order in which the iso-centres are delivered. Using a heuristic approach, the mean dose-rate has been used to explore the range of BED distributions achievable for a given plan. The induction rate of radiation damage is proportional to both the dose and the dose-rate at any given location inside the patient. However, the mean dose-rate across multiple voxels, as used to inform the re-sequencing, does not have a simple radiobiological interpretation.

These findings are in line with the observations from another publication aimed at quantifying the variation in the mean target BED with varying iso-centre sequences using a mono-exponential repair model [13]. However, the authors of this study found the original iso-centre sequence to be generally close to the ‘max BED’ sequence, a difference that could be explained with the fact that nearby iso-centres were fused together to reduce the number of possible permutations of the delivery sequence.

In practice, a maximised or minimised BED will not necessarily correlate with more beneficial treatment plans, e.g. where sparing nearby organs at risk (OARs) takes priority. For such cases, improving the BED-gradient may be more important. In general, optimising the iso-centre order for different scenarios during treatment planning will require a more sophisticated optimisation approach. Nevertheless, the variability in BED values for different sequences and the general feasibility of optimising these has now been demonstrated, an important step in the process of developing BED treatment planning for multi iso-centre treatments in GK radiosurgery. Such a planning system would initially seek to avoid BED ‘hot’ spots in the normal tissue at risk outside the margin of the lesion and at the same time also avoiding BED ‘cold’ spots in the lesion treated. In addition, it would mitigate the present patient to patient variation in the biologically effective dose, as has been proposed to limit complications to a reasonable level in the treatment of trigeminal neuralgia where only a single iso-centre is used [9]. Resolving the problems and complexities associated with inverse planning for multi iso-centre treatments using the bi-exponential BED model is beyond the scope of the current study.

### Limitations

The current study is limited to BED values on the prescription dose iso-surface to allow for the comparison of voxels receiving the same physical dose. Extending this analysis to the entire target volume is straightforward and leads to comparable results for the evaluation executed in this study.

The α/β ratio of 2.47 Gy used in the present investigation was derived as part of the best collective fit, along with the two repair half-times and a partition coefficient, to the data in the study by Pop et al [4]. The range of values covered by the 95% confidence interval was 1.50-3.95 Gy. Thus, a value of 2.47 Gy is not significantly different from the normal clinically accepted value of 2 Gy used routinely in radiotherapy practice. However, it has recently been shown [5] that the use of a range of α/β ratio values (1.5 – 3.0 Gy) does not significantly change the originally derived experimental relationship between the iso-effective dose for myelopathy and treatment time for a period of up to 10 hours, with the α/β-ratio effectively acting as a scaling factor. Millar et al. [8] also showed that the BED model with the previously obtained parameters, including the α/β ratio of 2.47 Gy, retrospectively provided a good fit to previously derived data for irradiated pig spinal cord, indicating that the repair kinetics are in fact species independent.

### BED calculation for treatment planning

The calculation of BED involves the consideration of many parameters that can vary in any GK treatment plan, i.e. dose/dose-rate and the timing of iso-centres and the intervals between iso-centres and can be carried out efficiently. The processing time in MATLAB for the calculation of a single 31×31×31 BED matrix from the original treatment parameters, as carried out by the research version of LGP for physical dose, does not exceed 0.05 *s*^§^ §Measured using the MATLAB built-in function timeit for a 20 iso-centre treatment plan (max number of iso-centres in cohort: 19) on a consumer-grade Laptop (Intel® Core™ i7-7700HQ, 16 GB RAM).

Thus, BED calculations during treatment planning could be implemented in a similar fashion to the present physical dose calculations to inform the treatment planner during manual planning. Alternatively, the BED could be evaluated and visualised for the final physical dose treatment plan.

Optimising the iso-dose sequence to achieve the most beneficial BED distribution is a more complex problem since it cannot be described by a continuous convex function. Given the observations made for the present cohort of cases, a first step could be to visualise the BED for a given iso-centre sequence and to then take a heuristic approach (e.g. pyramidal grouping so that the most effective iso-centres are in the middle of the treatment) when finalising the treatment plan. Any approach using BED for treatment planning will need to fix the ordering of iso-centres at the planning stage to ensure they are delivered in the sequence defined in the treatment plan. Any deviation in the delivered plan would result in a different BED from that intended.

Generally, the number, location, shape, and order of iso-centres to maximise the effectiveness, or minimise the probability of adverse effects, of a treatment would have to be optimised. Developing an optimisation tool in terms of the BED for GK treatment planning is a direction for future research.

## CONCLUSION

The present study has demonstrated how seemingly inconsequential gaps in treatment and the individual sequence of iso-centres can influence the biological effectiveness of any GK treatment. When significant gaps in the treatment delivery occur, in relation to the fast half-time for repair, there will be a reduction in the BED. The extent of the decrease will depend on the size and positioning of this gap, as well as the individual sequence of iso-centres for the treatment plan.

Ideally, the dose/dose-rate distribution in relation to location and timing should be considered during treatment planning to ensure consistency in the biological effectiveness of the radiation dose that is delivered. Handling the increased complexity is unlikely to be achieved with solely manual planning and will almost certainly require the use of an inverse planning method.
